# Clinical significance of blood cell ratios in healthy and sick *Leishmania infantum*-seropositive dogs

**DOI:** 10.1186/s13071-024-06522-z

**Published:** 2024-10-23

**Authors:** Giulia Donato, Marta Baxarias, Laia Solano-Gallego, Icíar Martínez-Flórez, Cristina Mateu, Maria Grazia Pennisi

**Affiliations:** 1 Università Di Messina, Messina, Italy; 2 ASC “I Periodeuti”, Reggio Calabria, Italy; 3 Departament de Medicina I Cirurgia Animal, Universitat Autònoma de Barcelona, Bellaterra, Spain; 4 Ecuphar Veterinaria SLU, Barcelona, Spain

**Keywords:** Canine leishmaniosis, Serum protein electrophoresis, Neutrophil-to-lymphocyte ratio, Monocyte-to-lymphocyte ratio, Platelet-to-lymphocyte ratio, Clinical staging and LeishVet

## Abstract

**Background:**

The accuracy of blood cell ratios (BCRs) as cost-effective and easily accessible diagnostic and prognostic markers of inflammatory conditions has been investigated in veterinary medicine in recent years.

**Methods:**

Neutrophil-to-lymphocyte (NLR), monocyte-to-lymphocyte (MLR), and platelet-to-lymphocyte (PLR) ratios were studied in 195 dogs clinically evaluated and tested for anti-*Leishmania infantum* (*Li*) antibodies (*Li*-seronegative (*Li*^−^), *n* = 10; *Li*-seropositive clinically healthy (*Li*^+^_healthy_), *n* = 100; *Li*-seropositive with clinical and/or clinicopathological abnormalities (*Li*^+^_sick_), *n* = 85). The *Li*^+^_sick_ dogs were classified in LeishVet stages IIa/IIb (*Li*^+^_IIa/IIb_) (*n* = 66) and III/IV (*Li*^+^_III/IV_) (*n* = 19). BCR relationships with LeishVet clinical stage, antibody levels, and serum protein electrophoretic fraction concentrations were investigated.

**Results:**

Higher NLR values were found in *Li*^+^, *Li*^+^_healthy_, and *Li*^+^_IIa/IIb_ sick dogs compared to *Li*^−^ dogs (*P* < 0.001). Higher NLR and MLR were found in *Li*^+^_sick_ (NLR, *P* < 0.001; MLR, *P* = 0.034) and *Li*^+^_III/IV_ dogs (NLR, *P* < 0.001; MLR, *P* = 0.005) compared to *Li*^−^ dogs, and in *Li*^+^_III/IV_ dogs (NLR, *P* = 0.002; MLR, *P* < 0.001) compared to *Li*^+^_healthy_. All three BCRs were higher in *Li*^+^_sick_ (NLR, MLR, *P* < 0.001; PLR, *P* = 0.023) and *Li*^+^_IIa/IIb_ dogs (NLR *P* < 0.001; MLR *P* = 0.001; PLR, *P* = 0.012) compared to *Li*^+^_healthy_ dogs. The BCRs failed to distinguish dogs with moderate (*Li*^+^_IIa/IIb_) and severe or very severe disease (*Li*^+^_III/IV_). BCRs demonstrated weak positive correlations with serum globulin fractions and antibody levels, and weak negative correlations with serum albumin level were found. *Li*^+^_sick_ dogs presenting hypoalbuminemia showed higher MLR ratios (*P* = 0.001) than those with normal albumin values.

**Conclusions:**

This study shows that BCR measures provide useful information for differentiating antibody-positive healthy and sick dogs at diagnosis. Dogs with hypoalbuminemia showed higher MLR values despite monocytosis being very rare.

**Graphical Abstract:**

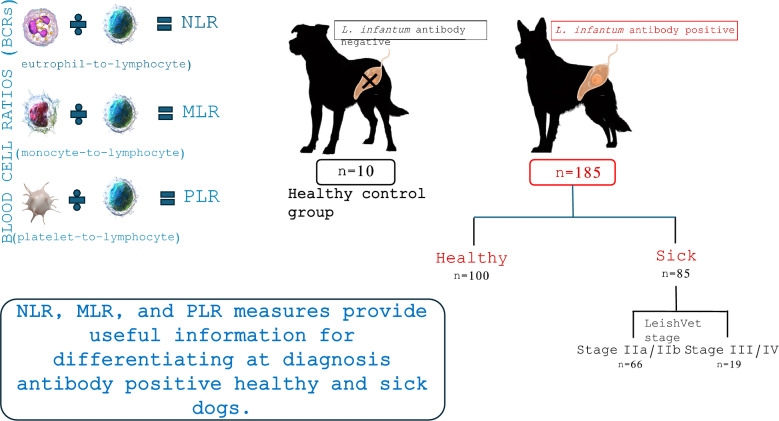

**Supplementary Information:**

The online version contains supplementary material available at 10.1186/s13071-024-06522-z.

## Background

Canine leishmaniosis (CanL) is a zoonotic disease caused by *Leishmania infantum* with a severe fatal course in some dogs [1]. Leishmaniosis is endemic in more than 70 countries, including those in Southern Europe, Northern Africa, the Middle East, Central Asia, China, and South America, and dogs represent the main domestic reservoir for *L. infantum* infection [2, 3]. The main route of transmission in endemic areas is vectorial, through the bite of female phlebotomine sand flies [1, 2]. However, other modes of transmission have been documented such as transplacental and venereal infections, or by transfusion of infected canine blood products. The non-vectorial transmission has a primary role in the epidemiology of foci of CanL in non-endemic areas where competent vectors are not present [1].

The course of CanL is influenced by the type of the dog immune response [4, 5]. In fact, the development of a progressive infection underlying the disease is associated with a marked humoral immune response and downregulation in host cell-mediated immunity [4, 5]. Individual dogs show different levels of both antibody and cellular adaptive immune responses. In endemic areas, most infected dogs are apparently healthy or have slight clinicopathological abnormalities, while others have a variably severe course of disease. Therefore, clinical staging systems are useful for treatment choice and to formulate prognosis [4]. The LeishVet clinical staging system takes into consideration the antibody level and the type of clinical signs and clinicopathological abnormalities detected [1].

Dogs with leishmaniosis may present various clinical signs and clinicopathological abnormalities that reflect an intense systemic inflammatory response. Markers of inflammation have been investigated primarily in dogs with clinical leishmaniosis, and increased levels of positive acute-phase proteins (APPs) such as serum ferritin [6–14], C-reactive protein (CRP) [6, 8–17], haptoglobin (Hp) [8, 11, 13–16], serum amyloid A (SAA) [8, 15, 16], and ceruloplasmin [8, 15] have been observed. Similarly, hypoalbuminemia and a decrease in other negative APPs such as transferrin (or total iron-binding capacity: TIBC) [6, 8] and paraoxonase 1 (PON-1) [8, 10, 11, 14] were reported. Increased levels of α_2_-globulins and γ-globulins were found in serum protein electrophoresis (SPE) analysis [8, 11, 13, 15, 17], and increases were found in the total level of immunoglobulins G (IgG) and M (IgM) [16].

The complete blood count (CBC) of dogs with clinical leishmaniosis may show a mild-normocytic normochromic non-regenerative anemia as a consequence of the chronic inflammation and sequestration of iron in macrophages; however, anemia can be moderate or severe in dogs with advanced chronic renal disease as additional patho-mechanisms occur [8]. White blood cell abnormalities are variable and may include neutrophilia, lymphopenia, lymphocytosis, and eosinophilia [1, 3, 8]. Moderate thrombocytopenia [8] or thrombocytosis [18] can also be detected.

There is great interest in cost-effective and easily accessible markers of inflammation because of their clinical relevance in prognosis and monitoring of diseases. Blood cell ratios (BCRs) have been extensively investigated with this aim in human medicine [19–36]. In dogs, BCRs have been examined in various infectious [37–40] and non-infectious [41–48] inflammatory conditions. Two studies [49, 50] evaluated BCRs in dogs with *L. infantum* infection comparing healthy and sick dogs [49] and assessing the prognostic potential of BCRs in dogs with chronic renal disease associated with *L. infantum* infection that were followed up [50].

This study considered some BCRs in *L. infantum* antibody-positive dogs, based on the hypothesis that differences may exist with *L. infantum* antibody-negative dogs and also between *L. infantum* antibody-positive healthy and sick dogs and among dogs with different severity of disease. With this aim, we studied neutrophil-to-lymphocyte (NLR), monocyte-to-lymphocyte (MLR), and platelet-to-lymphocyte (PLR) ratios in healthy seronegative dogs and *L. infantum* antibody-positive healthy and sick dogs. Specifically, we analyzed (a) differences between *L. infantum*-negative and seropositive dogs, (b) differences between *L. infantum* antibody-positive healthy and sick dogs, and (c) differences between dogs with different severity of leishmaniosis. Additionally, we analyzed (d) differences between *L. infantum* antibody-positive dogs presenting abnormalities in concentrations of the electrophoretic fractions and those with values within the reference intervals, and (e) relationships of BCRs with antibody levels and serum protein electrophoretic fraction concentrations.

## Methods

### Study description

A review of medical records of 185 apparently healthy dogs partly included in a previous published study was performed [51]. Specifically, data from 172 seropositive dogs (91 healthy dogs and 81 sick dogs) were from a multicentric study on the clinical status of *L. infantum* antibody-positive apparently healthy dogs in endemic areas [51]. Only dogs with CBC and SPE data available were selected. Among stage II sick dogs, only dogs substaged according to the measurement of proteinuria were included [1]. Data from an additional 13 *L. infantum* antibody-positive, apparently healthy dogs clinically evaluated by practitioners participating in the multicentric study were also included (nine healthy and four sick dogs). Ten beagles purchased from a breeder for research use (Isoquimen, Sant Feliu de Codines, Spain) were included as controls. They were housed indoors at UAB (Autonomous University of Barcelona) Veterinary School and were enrolled as control dogs because they were clinically healthy based on physical examination, CBC, biochemical profile with urinalysis and urine protein-to-creatinine ratio (UPC), SPE, and antibody negative to *L. infantum* antigen. Dogs were sampled between February 2020 and June 2021 in different areas of Spain (*n* = 161) and Italy (*n* = 34). Data regarding signalment, history, and physical examination findings were recorded. From the database of studied dogs, data related to their LeishVet clinical stage [1], CBC, SPE, and anti-*L. infantum* antibody levels were selected for the present study [51]. A total of 195 dogs (anti-*L. infantum* antibody-negative healthy dogs, *Li*^−^ *n* = 10; anti-*L. infantum* antibody-positive dogs, *Li*^+^ *n* = 185 including both healthy dogs, *Li*^+^_healthy_ *n* = 100 and dogs with clinical and/or clinicopathological abnormalities, *Li*^+^_sick_ *n* = 85) were studied. The *Li*^−^ group (median age: 24, range: 12–24 months; 25th–75th percentile = 15–24) included five male and five female Beagle dogs, classified as healthy seronegative according to physical examination and laboratory testing (CBC, SPE, biochemistry, and urinalysis with UPC). The *Li*^+^ dogs included more males (*Li*^+^_healthy_ *n* = 63; *Li*^+^_sick_, *n* = 53) than females (*Li*^+^_healthy,_ *n* = 37; *Li*^+^_sick_, *n* = 32), with 82 crossbreed and 103 purebred dogs of 34 different breeds (Supplementary Table 1). The age range was 12–168 months in *Li*^+^_healthy_ dogs (median age = 48 months; 25th–75th percentile = 36–75) and 5–144 months in *Li*^+^_sick_ dogs (median age = 60 months; 25th–75th percentile = 36–96). The *Li*^+^_sick_ dogs were classified according to the LeishVet clinical staging system [1], and two groups of staged dogs were considered for statistical analysis: stage IIa/IIb group (*Li*^+^_IIa/IIb_, *n* = 66) including dogs with moderate disease and the stage III/IV group (*Li*^+^_III/IV_, *n* = 19) including dogs with severe/very severe disease.

### Clinicopathological and serological evaluation

The CBC was performed using the XN-1000 analyzer (Sysmex España SL, Sant Just Desvern, Spain) or Advia 2120 (Siemens Healthcare SRL, Milan, Italy), and blood smears were also examined for cell morphological abnormalities, detection of hemoparasites, and to exclude samples from the statistical analysis when platelet clumps were observed. The absolute concentrations of lymphocytes, neutrophils, monocytes, and platelets were evaluated. Neutrophil, monocyte, and platelet values were divided by absolute concentrations of lymphocytes, and neutrophil-to-lymphocytes (NLR), monocytes-to-lymphocytes (MLR), and platelet-to-lymphocyte (PLR) ratios were calculated. The PLR was calculated in overall 100 dogs, as in 95 dogs platelet aggregates were detected in blood smears and platelet concentration could not be used. The SPE was evaluated using the Capillarys 3 (Sebia Dubai SA, Dubai, UAE), and reference intervals are reported in Supplementary Table S2. An in-house enzyme-linked immunosorbent assay (ELISA) was performed on the sera of all dogs studied for the detection of anti-*Leishmania* antibodies as previously described [51]. The result was quantified as ELISA units (EU) and sera were classified as high positive when having a positivity percentage equal to or higher than 300 EU, medium positive when having a positive percentage equal to or higher than 150 EU and less than 300 EU, and low positive when having a positivity percentage lower than 150 EU and equal to or higher than 35 EU [51]. ELISA endpoint values were measured in all samples classified as medium or high positive, performing twofold serial dilutions [51].

### Statistical analysis

Statistical analysis was performed using Jamovi 2.3.28.0 statistical software. The distribution of continuous variables was evaluated by the Shapiro–Wilk test and descriptive statistics were obtained for all the investigated variables.

The Mann–Whitney *U*-test was used to evaluate differences in endpoint ELISA levels, SPE fractions, and BCRs between groups of dogs as follows: *Li*^−^ vs. *Li*^+^, *Li*^−^ vs. *Li*^+^_healthy_, *Li*^−^ vs. *Li*^+^_sick_, *Li*^+^_healthy_ vs. *Li*^+^_sick_, *Li*^−^ vs. *Li*^+^_IIa/IIb_, *Li*^−^ vs. *Li*^+^_III/IV_, *Li*^+^_healthy_ vs. *Li*^+^_IIa/IIb_, *Li*^+^_healthy_ vs. *Li*^+^_III/IV_, *Li*^+^_IIa/IIb_ vs. *Li*^+^_III/IV_. Similarly, the Mann–Whitney *U*-test was used to evaluate differences in lymphocyte, neutrophil, monocyte, and platelet concentrations among *Li*^−^ and *Li*^+^, *Li*^+^_healthy_ and *Li*^+^_sick_, *Li*^+^_healthy_ and *Li*^+^_IIa/IIb_, *Li*^+^_healthy_ and *Li*^+^_III/IV_, *Li*^+^_IIa/IIb_ and *Li*^+^_III/IV_. The number of *Li*^+^_sick_ dogs with out-of-range lymphocyte, monocyte, neutrophil, and platelet concentrations was evaluated, and the prevalence in *Li*^+^_IIa/IIb_ and *Li*^+^_III/IV_ dogs was compared by Fisher’s exact test.

Spearman’s rho test was used to measure the strength of the correlations between NLR, MLR, and PLR values and SPE fractions in the total cohort and endpoint ELISA levels in the *Li*^+^ dogs. The strength of this relationship, according to the correlation coefficient absolute value (*r*_s_), was qualified as follows: *r*_s_ = 1: perfect correlation; 1 > *r*_s_ ≥ 0.8: strong correlation; 0.8 > *r*_s_ ≥ 0.4: moderate correlation; 0.4 > *r*_s_ > 0.141 (NLR, MLR) or > 0.199 (PLR): weak correlation; *r*_s_ < 0.141 (NLR, MLR) or < 0.199 (PLR): no correlation [52]. The critical value of *r*_s_ was established on the basis of the number of degrees of freedom for each parameter evaluated [53]. Differences were considered significant if *P*-values were < 0.05.

## Results

Descriptive statistics and significant Mann–Whitney *U*-test of ELISA levels, NLR, MLR, PLR, and SPE fractions results are presented in Table 1. All three BCRs were higher in *Li*^+^_sick_ and *Li*^+^_IIa/IIb_ dogs compared to *Li*^+^_healthy_ dogs. Higher NLR and MLR were found in *Li*^+^_sick_ and *Li*^+^_III/IV_ dogs compared to *Li*^−^ dogs and in *Li*^+^_III/IV_ dogs compared to *Li*^+^_healthy_ dogs. *Li*^−^ dogs had significantly lower NLR values than any category of antibody-positive dogs considered, except for *Li*^+^_III/IV_ dogs.

**Table 1:** Descriptive statistics of blood cell ratios, ELISA levels (ELISA units), and serum electrophoretic fractions (g/l) for the enrolled dogs

	*Li*^−^ Median (Min–Max) [25th–75th] (*n* = 10)	*Li*^+^ Median (Min–Max) [25th–75th] (*n* = 185)	*Li*^+^_healthy_ Median (Min–Max) [25th–75th] (*n* = 100)	*Li*^+^_sick_ Median (Min–Max) [25th–75th] (*n* = 85)	*Li*^+^_IIa/IIb_ Median (Min–Max) [25th–75th] (*n* = 66)	*Li*^+^_III/IV_ Median (Min–Max) [25th–75th] (*n* = 19)	Mann–Whitney *U*-test *P*
NLR	1.49 (1.05–3.91) [1.28–1.73]^A,B,C,E,F^	3.1 (1–48) [2.3–4.5]^A^	2.8 (1.3–30) [2.1–3.8]^B,D,G,H^	3.7 (1–48) [2.6–5.7]^C,D^	3.7 (1–48) [2.52–5.78]^E,G^	4 (1.6–14.3) [3.3–5.55]^F,H^	< 0.001^A,B,C,D,E,F,G^ 0.002^H^
MLR	0.18 (0.12–0.35) [0.15–0.22]^C,F^	0.2 (0.0–1.3) [0.1–0.4]	0.2 (0.1–1.3) [0.1–0.3]^D,G,H^	0.3 (0–1.2) [0.2–0.4]^C,D^	0.3 (0–1.2) [0.2–0.4]^G^	0.3 (0.1–1.1) [0.3–0.5]^F,H^	0.001^G^ < 0.001^D,H^ 0.005 ^F^ 0.034^C^
PLR	123 (60.4–217) [94.1–160]	123 (17.2–1290) [77.3–182]	107 (40.5–267) [73.5–158]^D, G^	150 (17.2–1290) [87.9–197]^D^	163 (31.8–1290) [101–197]^G^	136 (17.2–484) [82.4–193]	0.012^G^ 0.023^D^
ELISA	5.79 (4.42–7.65) [4.97–6.24]	241 (4–11,114) [119–813]	142 (4–1210) [101–250]^D,G,H^	752 (4.4–11,114) [197–2933]^D^	616 (87.2–8594) [187–2136]^G^	1794 (4.4–11,114) [281–3405]^H^	< 0.001^D,G,H^
Albumin	32.5 (29.7–35.7) [31.8–33.3]^B,F^	34.6 (15.3–49.1) [31.1–37.8]	36.5 (25.5–45.4) [34.3–39]^B,D,G,H^	31.6 (15.3–49.1) [27.1–34.6]^D^	32.9 (21.2–49.1) [28.8–35.3]^G,I^	27.3 (15.3–34.1) [22.6–30.7]^F,H,I^	< 0.001^B,D,F,G,H,I^
α_1_-Globulin	3.45 (3–4) [3.18–3.65]	3.5 (1.7–10.2) [3, 4]	3.4 (1.7–4.8) [2.9–3.73]^D,G,H^	3.7 (2–10.2) [3.2–4.3]^D^	3.7 (2–5.9) [3.2–4.27]^G^	3.7 (2.8–10.2) [3.25–4.6]^H^	0.008^G^ 0.002^D^ 0.013^H^
α_2_-globulin	6.9 (5.3–8.6) [5.95–7.38]^C,E,F^	7.2 (2.8–18.1) [6.2–8.7]	6.5 (2.8–12.7) [5.7–7.5]^D,G,H^	8.4 (4.5–18.1) [7.1–9.5]^C,D^	8.45 (4.9–18.1) [7.23–9.17]^E,G^	8.1 (4.5–13.6) [6.85–11]^F,H^	< 0.001^D,G,H^ 0.004^C,E^ 0.020 ^F^
β-globulin	7.1 (6.5–8.3) [6.95–7.73]^A,B,C,E,F^	12.9 (1.4–37.9) [11.4–15.4]^A^	12.1 (1.4–24.3) [10.4–14.2]^B,D,G,H^	14.1 (9.2–37.9) [12.1–17.7]^C,D^	13.9 (9.2–37.9) [12.1–16.9]^E,G^	15.3 (10.3–24.8) [13.1–19.9]^F,H^	< 0.001^A,B,C,D,E,F,G,H^
γ-globulin	3.45 (2.6–4.7) [3.2–4.17]^A,B,C,E,F^	9.8 (4.4–56) [7.7–14.1]^A^	8.2 (4.4–13.7) [7–9.4]^B,D,G,H^	14.5 (5.3–56) [11–21.6]^C,D^	14.1 (5.3–50.8) [11–19]^E,G,I^	22.1 (8.6–56) [12.3–30]^F,H,I^	< 0.001^A,B,C,D,E,F,G,H^ 0.024^I^

*Min* minimum, *Max* maximum, *25th*  25th percentile, *75th* 75th percentile, *P* *P*-values, *NLR* neutrophil-to-lymphocyte ratio, *MLR* monocyte-to-lymphocyte ratio, *PLR* platelet-to-lymphocyte ratio, *Li*^−^ anti-*L. infantum* antibody-negative healthy dogs, *Li*^+^ anti-*L. infantum* antibody-positive dogs, *Li*^+^_healthy_ *Li*-seropositive healthy dogs, *Li*^+^_sick_ dogs with clinical and/or clinicopathological abnormalities, *Li*^+^_IIa/IIb_ dogs in LeishVet stage IIa/IIb, *Li*^+^_III/IV_ dogs in LeishVet stage III/IV. ^§^ = PLR was calculated in 100 dogs, 7 *Li*^*−*^*,* 54 *Li*^+^_healthy_, 39 *Li*^+^_sick_, 27 *Li*^+^_IIa/IIb_ dogs, and 12 *Li*^+^_III/IV_ dogs respectively. Endpoint ELISA results were evaluated for each group of *Li*^+^ dogs. Significant comparisons: A = *Li*^+^  > *Li*^−^; B = *Li*^+^_healthy_ > *Li*^−^; C = *Li*^+^_sick_ > *Li*^−^; D = *Li*^+^_sick_ > *Li*^+^_healthy_; E = *Li*^+^_IIa/IIb_ > *Li*^−^; F = *Li*^+^_III/IV_ > *Li*^−^; G = *Li*^+^_IIa/IIb_ > *Li*^+^_healthy_; H = *Li*^+^_III/IV_ > *Li*^+^_healthy_; I = *Li*^+^_III/IV_ > *Li*^+^_IIa/IIb_

Descriptive statistics and significant Mann–Whitney *U*-test results for lymphocyte, neutrophil, monocyte, and platelet concentrations are shown in Supplementary Table 3 (Table S3), and the number of *Li*^+^_sick_ dogs with out-of-range values of these blood cell concentrations are reported in Supplementary Table 4 (Table S4).

Correlations of BCRs with ELISA and SPE results evaluated in the total cohort are described in Table 2. BCRs demonstrated weak positive correlations with serum globulin fractions and antibody levels, and weak negative correlations with serum albumin level were found. *Li*^+^_sick_ dogs with hypoalbuminemia had higher MLR (median = 0.450; range = 0.3–0.7; 25th–75th percentile = 0.325–0.575) (*P* = 0.001) than those with albumin values within the reference interval (median = 0.3; range = 0.0–1.2; 25th–75th percentile = 0.2–0.4). No other differences were found concerning the other electrophoretic fractions, and NLR and PLR.

**Table 2:** Spearman’s rho test between neutrophil-to-lymphocyte (NLR), monocyte-to-lymphocyte (MLR), platelet-to-lymphocyte (PLR) ratios, and endpoint ELISA or serum protein electrophoresis in the total cohort

		NLR		MLR		PLR	
Albumin	*r*_s_	−0.150*		–0.369*		−0.159	
	*P*	0.036		< 0.001		0.114	
α_1_-Globulins	*r*_s_	0.158*		0.184*		0.244*	
	*P*	0.027		0.010		0.014	
α_2_-Globulins	*r*_s_	0.361*		0.309*		0.299*	
	*P*	< 0.001		< 0.001		0.003	
β-Globulins	*r*_s_	0.143*		0.076		−0.081	
	*P*	0.047		0.294		0.425	
γ-Globulins	*r*_s_	0.306*		0.276*		0.116	
	*P*	< 0.001		< 0.001		0.251	
ELISA^a^	*r*_s_	0.185*		0.294*		0.248*	
	*P*	0.012		< 0.001		0.017	

*r*_s_ = Spearman’s rho. * Significant difference. ^a^ Spearman’s rho test between NLR, MLR, PLR, and ELISA levels was evaluated only in *Li*^+^ dogs

## Discussion

The purpose of this study was to evaluate selected BCRs (NLR, MLR, and PLR) as markers for differentiating *L. infantum* antibody-positive healthy and sick dogs and for staging the severity of disease in sick dogs. We found higher NLR values in *Li*-seropositive compared to *Li*-seronegative dogs. Interestingly, in the *Li*^+^ sick dogs ,the NLR, MLR, and PLR values were higher than in *Li*^+^ healthy dogs, and (excluding the PLR) in *Li*-seronegative animals as well. Furthermore, *Li*^+^ sick dogs presenting hypoalbuminemia showed higher MLR ratios than animals with normal albumin values.

Clinically staged sick dogs with both severe/very severe (stages III/IV) and moderate (stages IIa/IIb) disease had higher NLR values than *Li*^+^ healthy and *Li*^−^ dogs. However, NLR failed to distinguish dogs between moderate (stages IIa/IIb) and severe or very severe disease (stages III/IV). Stage II is the more frequently observed LeishVet stage in dogs receiving a diagnosis of clinical leishmaniosis in endemic areas and the number of sick dogs with severe or very severe disease was low because all the studied dogs were from a population of apparently healthy dogs from *L. infantum*-endemic areas [4, 51]. Moreover, LeishVet stage I dogs were not studied as they have mild clinical signs and can be antibody negative, while we considered data from antibody-positive apparently healthy dogs*,* and this is a limitation for the aim to consider BCRs as markers of disease severity [1, 4, 51, 54, 55]. However, significant correlations of BCRs with two markers of disease severity (high antibody levels and abnormalities in SPE fractions) were found (Table 2) [1, 4]. As could be expected, hypoalbuminemia and increases in γ-globulins were confirmed to be markers of disease severity, as their values were significantly different among dogs with moderate and severe disease (Table 1).

Only two previous investigations have evaluated BCR values in CanL. Ferreira et al. (2021) compared NLR values among symptomatic and asymptomatic *L. infantum*-positive dogs and with *L. infantum*-negative control dogs [49]. They reported higher values in symptomatic than in asymptomatic dogs and in both groups compared to control dogs, in agreement with the present results [49]. Duran-Galea et al. (2024) focused on the prognostic value of NLR and PLR in leishmaniotic dogs with chronic kidney disease (CKD) staged according to the Immune Reconstitution Inflammatory Syndrome (IRIS) stage system [50]. Interestingly, they reported that the progression of CKD was positively correlated with NLR values and a short-term fatal course of disease [50]. In the present study, the NLR was useful for differentiating seropositive healthy and sick dogs and correlated with markers of disease severity.

BCRs as diagnostic and prognostic markers of various inflammatory conditions in dogs have been more extensively investigated [37–45]. In CanL, both acute and chronic inflammation may occur [56, 57], and increases in neutrophil and monocyte concentrations can be observed [58]. Neutrophilia and monocytosis were both rarely found in the present study; however, the group of dogs with more severe disease had significantly higher values of monocytes than *Li*^+^_healthy_ dogs. Conversely, lymphopenia contributed to the increases in BCRs observed in the *Li*^+^_sick_ dogs and it occurred in dogs with both moderate and severe disease, likely due to stress leukogram. Importantly, we found no cases of lymphocytosis that are reported in mild forms of CanL [59]. The relationship between NLR and hypoalbuminemia has been investigated in other inflammatory canine diseases [41, 44]. Benvenuti et al. (2020) evaluated NLR values in dogs affected by inflammatory bowel disease (IBD) and found a negative correlation between NLR and albumin values [44]. Becher et al. (2021) reported the same result in dogs with chronic enteropathy [41]. To the best of our knowledge, no data are available in the literature on the relationship between NLR, MLR, and PLR values with the other serum electrophoretic fractions.

In the present study, PLR values of the total cohort were positively correlated with ELISA and α-globulin measures (Table 2), and they differentiated healthy and sick dogs (Table 1). Other differences were not found, and we have to consider that this ratio was obtained in a lower number of dogs (*n* = 100) compared to NLR and MLR (*n* = 195), as PLR values were not calculated when platelets clumps were observed in blood smears. This is a limitation for the feasibility of platelet concentration that may occur in practice. Additionally, thrombocytopenia was more frequent than thrombocytosis, and significantly higher numbers of platelets were observed only in dogs with moderate disease versus *Li*^+^_healthy_ dogs. From the overall evaluation of the significant correlations, it appears that NLR and MLR could be more useful in the clinicopathological evaluation of disease severity.

Reference intervals for NLR, MLR, and PLR in healthy dogs are not currently available, so their use in routine practice is not generally performed. However, some studies have proposed reference intervals for NLR in healthy control dogs, using different methodologies, and reporting vastly different upper limits: 10.91 [38] and 4.1 [41]. Defining a reference interval for healthy dogs was not one of the aims of the present study; however, our data add more information to the literature. The upper limit for NLR recorded in negative healthy dogs of the present study (3.91) was similar to that reported by Becher et al. (2021) [41]. Conversely, no published data are available on MLR and PLR upper limits in healthy dogs; therefore, comparisons with other results are not possible, and additional large studies are needed to establish reference intervals.

We considered these data preliminary, as we only evaluated the relationship between some BCRs and changes in serum electrophoretic fractions. Other BCRs and the relationship among BCRs and additional clinicopathological parameters were not determined. Apart from the relationship of BCRs with other CBC, biochemical, and urinary data, those with APP changes currently available from commercial laboratories and studied in dogs with leishmaniosis could be considered in further studies [6–17]. Breed-related differences in absolute concentrations of white blood cells and platelets were not recognized among the pure breeds of enrolled dogs. The demographic data for studied dogs could be a limitation, as control dogs were all beagles and significantly younger than the antibody-positive dogs. Moreover, no beagle breed dog was among the *L. infantum* antibody-positive dogs. Bourgès-Abella et al. [60] found moderate differences between the reference intervals obtained in a large group of laboratory beagles and those previously reported for various breeds [60]. However, Kimura and Kotani [61] did not find age-related variations in white blood cell and platelet concentrations in beagles from 6 to 60 months of age [61].

Validation of the BCRs as diagnostic and prognostic markers of CanL is of great practical interest, particularly when clinical decisions have to be made on the basis of cost-effective and easily accessible diagnostic investigations. The BCRs are easily calculated from the CBC report and are available with no additional blood tests. This means that no additional blood volume is required, and no extra costs are charged to owners. In fact, apart from some patients with critical conditions, the CBC is always included in the minimal clinicopathological evaluation database of dogs, in toy breed dogs, and in cases of severe anemia when the volume of blood taken is restricted. The overall results of this study support the hypothesis that the studied BCRs could be an additional marker for CanL.

## Conclusions

The BCRs measured provided useful information for differentiating antibody *L. infantum*-positive healthy and sick dogs, but with the limitations of the present study, a clear differentiation among dogs with different severity of disease was not possible. However, dogs with hypoalbuminemia showed higher MLR values despite the fact that monocytosis was very rare.

## Supplementary Information

Supplementary Material 1.

## Data Availability

No datasets were generated or analyzed during the current study.
