# Supplementary material for: Clinical significance of blood cell ratios in healthy and sick Leishmania infantum-seropositive dogs
Source: Parasit Vectors. 2024 Oct 23;17:435. doi: 10.1186/s13071-024-06522-z (PMC11515770; doi:10.1186/s13071-024-06522-z)
Supplement: Supplementary file 1 — Supplementary Material 1. [file 13071_2024_6522_MOESM1_ESM.docx]

**Table S1**. Reference intervals for serum protein electrophoresis (SPE) parameters evaluated with Capillarys 3, Sebia Dubai SA, Dubai, UAE (Italy, Spain). Values were considered outside the reference interval if they were above or below the 15% of the upper or lower reference limit, respectively.

| SPE parameter (g/L) | Capillarys 3, Sebia |
| --- | --- |
| Albumin | 24.4-49.6 |
| α_1_-globulins | 1.7-4.5 |
| α_2_-globulins | 3.8-10.2 |
| β-globulins | 8-18 |
| γ-globulins | 2.6-11.7 |

**Table S2**. Breed distribution in 10 control dogs (healthy *L. infantum* antibody negative dogs), 100 *L. infantum* antibody positive healthy and 85 *L. infantum* antibody positive sick dogs.

| Dog breeds | Control | *L. infantum* antibody positive healthy | *L. infantum* antibody positive sick |
| --- | --- | --- | --- |
| Cross breed | 0 | 46 | 36 |
| Ibizian hound | 0 | 11 | 3 |
| Beagle | 10 | 0 | 0 |
| Labrador retriever | 0 | 5 | 2 |
| German shepherd | 0 | 4 | 3 |
| Jack Russel | 0 | 4 | 1 |
| Weimaraner | 0 | 3 | 1 |
| English setter | 0 | 3 | 2 |
| American pit bull | 0 | 2 | 2 |
| American Staffordshire | 0 | 2 | 3 |
| Border collie | 0 | 2 | 2 |
| Boxer | 0 | 2 | 1 |
| Spanish sighthound | 0 | 2 | 3 |
| Segugio Italiano | 0 | 2 | 0 |
| Spanish alano | 0 | 2 | 1 |
| Andalusian wine-cellar rat-hunting dog | 0 | 2 | 3 |
| Akita inu | 0 | 1 | 0 |
| American bully | 0 | 1 | 1 |
| Corso Italiano | 0 | 1 | 0 |
| Newfoundland | 0 | 1 | 0 |
| Pachon navarro | 0 | 1 | 0 |
| Rottweiler | 0 | 1 | 2 |
| Segugio Maremmano | 0 | 1 | 0 |
| Breton spaniel | 0 | 1 | 1 |
| Spanish mastiff | 0 | 0 | 3 |
| Andalusian hound | 0 | 0 | 2 |
| Argentine dogo | 0 | 0 | 2 |
| German hound | 0 | 0 | 2 |
| Spanish hound | 0 | 0 | 2 |
| Majorca shepherd | 0 | 0 | 1 |
| Chihuahua | 0 | 0 | 1 |
| Dachshund | 0 | 0 | 1 |
| Jagdterrier | 0 | 0 | 1 |
| Pointer | 0 | 0 | 1 |
| Presa canario | 0 | 0 | 1 |
| Yorkshire | 0 | 0 | 1 |

**Table S3.** Descriptive statistics and significant Mann-Whitney U test results of neutrophil, lymphocyte, monocyte, and platelet counts

|  | *Li*^-^  Median (Min-Max)  [25^th^-75^th^]  (n=10) | *Li^+^*  Median (Min-Max)  [25^th^-75^th^]  (n=185) | *Li*^+^_healthy_  Median (Min-Max)  [25^th^-75^th^]  (n=100) | *Li*^+^_sick_  Median (Min-Max)  [25^th^-75^th^]  (n=85) | *Li*^+^_IIa/IIb_  Median (Min-Max)  [25^th^-75^th^]  (n=66) | *Li*^+^_III/IV_  Median (Min-Max)  [25^th^-75^th^]  (n=19) | Mann-Whiteny U test  *p* |
| --- | --- | --- | --- | --- | --- | --- | --- |
| Neutrophils | 4795 (3810-9620)  [4355-5195]^A,B,C,E^ | 5999 (2394-20002)  [4935-7792]^A^ | 5881 (2714-14805)  [4937-7248]^B^ | 6263 (2394-20002)  [4910-8504]^C^ | 6344 (2394-20002)  [4998-8569]^E^ | 6135 (2633-10926)  [4597-8059] | 0.022^A,E^  0.026^B^  0.027^C^ |
| Lymphocytes | 2890 (1960-4580)  [2658-3493]^A,B,C,E,F^ | 1924 (235-4299)  [1448-2549]^A^ | 2091 (358-4299)  [1648-2783]^B,D,G,H^ | 1711 (235-4080)  [1142-2265]^C,D^ | 1712 (235-4080)  [1205-2249]^E,G^ | 1705 (616-3376)  [987-2189]^F,H^ | < 0.001^A,C,D,E,F^  0.002^B,G^  0.011^H^ |
| Monocytes | 530 (420-880)  [470-678]^B^ | 437 (0-2047)  [298-622] | 423 (128-1269)  [295-571]^B,H^ | 459 (0-2047)  [322-725] | 438 (0-2047)  [313-683] | 611 (171-1447)  [354-749]^H^ | 0.026^B^  0.037^H^ |
| Platelets (10^3^  /µL) | 371 (253-994)  [309-440]^A,B,C,E,F^ | 253 (40-630)  [178-309]^A^ | 239 (101-471)  [178-292]^B,G^ | 268 (40-630)  [203-328]^C^ | 282 (62-630)  [230-330]^E,G^ | 254 (40-379)  [141-311]^F^ | 0.005^A^  0.003^B^  0.023^C^  0.040^E^  0.025^F^  0.038^G^ |

Legend: Min= minimum; Max= maximum; 25^th^ = 25^th^ percentile; 75^th^= 75^th^ percentile; *p*= p values; *Li*^-^=anti-*L. infantum* antibody negative healthy dogs; *Li*^+^= anti-*L. infantum* antibody positive dogs; *Li*^+^_healthy_= *Li* antibody positive healthy dogs; *Li*^+^_sick_= *Li* antibody positive dogs with clinical and/or clinical-pathological abnormalities; *Li*^+^_IIa/IIb_= *Li*^+^sick dogs in LeishVet stage IIa/IIb; *Li*^+^_III/IV_= *Li*^+^sick dogs in LeishVet stage III/IV. Significant comparisons: ^A^*=* *Li*^+^ > *Li*^-^; ^B^*=Li*^+^_healthy_ > *Li*^-^; ^C^= *Li*^+^_sick_> *Li*^-^; ^D^= *Li*^+^_sick_ > *Li*^+^_healthy_; ^E^= *Li*^+^_IIa/IIb_ > *Li*^-^; ^F^= *Li*^+^_III/IV_ > *Li*^-^; ^G^= *Li*^+^_IIa/IIb_ > *Li*^+^_healthy_; ^H^= *Li*^+^_III/IV_ > *Li*^+^_healthy_; ^I^= *Li*^+^_III/IV_ > *Li*^+^_IIa/IIb._

**Table S4.** Number (n) of dogs with neutrophil, lymphocyte, monocyte, and platelet values outside the reference range (RR) in the overall 85 *L. infantum* antibody-positive sick dogs, and in stage IIa-IIb or III-IV sick dogs. Values were considered outside the reference range if they were above or below the 15% of the upper or lower reference limit, respectively. No significant differences (Fisher’s Exact test) were found among values outside the RR when 66 stage IIa/IIb and 19 stage III/IV dogs were compared.

| Parameter (RR) | *L. infantum* antibody-positive sick dogs  n (%) | Stage IIa/IIb  n (%) | Stage III/IV  n (%) |
| --- | --- | --- | --- |
| Neutrophils (2900–13600/µL) |  |  |  |
| Neutropenia | 2 (2.3) | 1 (1.5) | 1 (5.3) |
| Neutrophilia | 5 (5.9) | 3 (4.5) | 2 (10.5) |
| Lymphocytes (1100–5300/µL) |  |  |  |
| Lymphopenia | 14 (16.5) | 9 (13.6) | 5 (26.3) |
| Monocytes (400–1600/µL) |  |  |  |
| Monocytopenia | 22 (25.9) | 18 (27.3) | 4 (21.1) |
| Monocytosis | 2 (2.3) | 1 (1.5) | 1 (5.3) |
| Platelets (200–500 10^3^/µL)^^^ |  |  |  |
| Thrombocytopenia | 8 (20.5) | 4 (14.8) | 4 (33.3) |
| Thrombocytosis | 2 (5.1) | 2 (7.4) | 0 |

Legend: ^^^ platelet count was available in overall 39 *L. infantum* antibody-positive sick dogs (27 stage II-III dogs and 12 stage III-IV dogs respectively).
